# Lens Thickness Microfluctuations in Young and Prepresbyopic Adults During Steady-State Accommodation

**DOI:** 10.1167/iovs.64.2.12

**Published:** 2023-02-08

**Authors:** Leana Rohman, Marco Ruggeri, Arthur Ho, Jean-Marie Parel, Fabrice Manns

**Affiliations:** 1 Ophthalmic Biophysics Center, Bascom Palmer Eye Institute, University of Miami Miller School of Medicine, Miami, Florida, United States; 2 Department of Biomedical Engineering, University of Miami College of Engineering, Coral Gables, Florida, United States; 3 Brien Holden Vision Institute, Sydney, New South Wales, Australia; 4 School of Optometry and Vision Science, University of New South Wales, Sydney, New South Wales, Australia

**Keywords:** accommodation, presbyopia, mechanics, lens, imaging, OCT, optical coherence tomography, lens thickness, heart rate

## Abstract

**Purpose:**

To determine whether lens mechanical dynamics change with age and with accommodative demands.

**Methods:**

Lens thickness microfluctuations were measured using a high-speed custom-built spectral domain optical coherence tomography system in five young adults (20 to 25 years old) at 0 diopters (D), 2 D, 4 D, and maximum accommodative demand and in five prepresbyopes (38 to 45 years old) under relaxed and maximal accommodation. For each state, the measurements were repeated four times during the same session. Images of the central 2-mm zone of the lens comprising 170 A-lines/frame were acquired for 10 seconds, and axial lens thickness change was measured. Lens thickness microfluctuations (µm²/Hz) were assessed by integrating the power spectrum of lens thickness microfluctuations between 0 and 4 Hz.

**Results:**

The amplitude of lens microfluctuations was higher in the accommodated states than in the relaxed state in both age groups. Lens microfluctuations were higher in young adult participants than in prepresbyopes, with a significant difference in relaxed and maximally accommodated states (*P* = 0.04 and *P* = 0.04). In the young participants, the amplitude of microfluctuations reached a plateau at maximum accommodation.

**Conclusions:**

Lens mechanical dynamics are both age and accommodation dependent. The decrease in lens thickness microfluctuations with age is consistent with an age-related increase in lens stiffness or decrease of the ciliary muscle displacement. The lens does not contribute to the high-frequency component of ocular dioptric microfluctuations.

Studies starting in the mid-20th century have shown that ocular power fluctuates when viewing a stationary target.^1^ Prior research has suggested that these fluctuations, commonly known as microfluctuations of accommodation, have two dominant components: a low-frequency component (LFC) below 0.6 Hz and a high-frequency component (HFC) between 1.0 Hz and 2.5 Hz.^1^^–^^3^ The origin of these two components, however, remains a matter of contention. These fluctuations may reflect the neural control mechanism to maintain focus or mechanical instability in the lens caused by changes in zonular tension. Some authors have suggested that the LFC is an inherent part of the accommodative control loop since its response is influenced by the task and conditions of observation.^4^^–^^7^ Although no consensus has yet been reached concerning the origin of the HFC, the arterial pulse is often cited as the source of these high temporal frequencies.^8^^,^^9^

It is reasonable to assume that microfluctuations of accommodation are due in part to fluctuations in lens shape since changes in lens shape are responsible for ocular power changes during accommodation. Quantifying fluctuations in lens shape can therefore provide valuable insight into the origins of the LFC and HFC and their role in the accommodative response. In addition, fluctuations in lens shape are also a mechanical characteristic of the dynamic accommodative response. Quantifying the age dependence in lens shape fluctuations may therefore help improve our understanding of the changes in lens mechanical properties in relation to presbyopia.

There have been only a few studies regarding the mechanical origins of microfluctuations of accommodation.^10^^,^^11^ Using dynamic A-mode ultrasonography, Van der Heijde et al.^10^ quantified microfluctuations in ocular biometry during steady-state accommodation. In a study on seven young adults, they found that the LFC is associated with fluctuations in lens shape and that the HFC is a consequence of fluctuations in axial eye length. Furthermore, they observed that LFC fluctuations increased with increasing accommodation, up to 4 diopters (D) accommodation stimulus, and then decreased to the initial level with larger accommodations. A limitation of ultrasonography is that it requires contact with the eye. The pressure applied to the globe by the probe may influence some of the measured fluctuations on ocular dimensions, particularly in eye length. More recently, Gambra et al.^11^ demonstrated the feasibility of recording fluctuations in lens curvature and thickness at steady-state accommodation with optical coherence tomography (OCT) in a study on four young adults. They found that the HFC of lens shape fluctuations is larger than the LFC at all accommodative demands and that fluctuations in lens shape increase as the accommodation demand increases, mostly due to an increase in the HFC. These findings contradict the results of the earlier study by Van der Heijde et al.,^10^ who found an absence of HFC in the lens thickness fluctuations. The discrepancy may be due to differences in methodology used for ocular biometry (ultrasound versus OCT).

The purpose of the present study is to quantify lens thickness microfluctuations at steady state of accommodation using high-speed OCT biometry and to determine if they change with accommodation and with age.

## Methods

### Human Participants

Two groups of participants were enrolled prospectively: (1) five young adults, aged 20 to 25 years, and (2) five prepresbyopes, aged 38 to 45 years. In young participants, the mean spherical equivalent refraction ranged from −0.38 to −3.38 D, while in prepresbyopes, it ranged from −0.75 to −4.38 D. The study was approved by the University of Miami Institutional Review Board and followed the tenets of the Declaration of Helsinki. All participants provided written informed consent. Inclusion criteria included the ability to maintain steady fixation on the accommodative target.

### Spectral Domain OCT Imaging and Photoplethysmography

**Figure 1.**
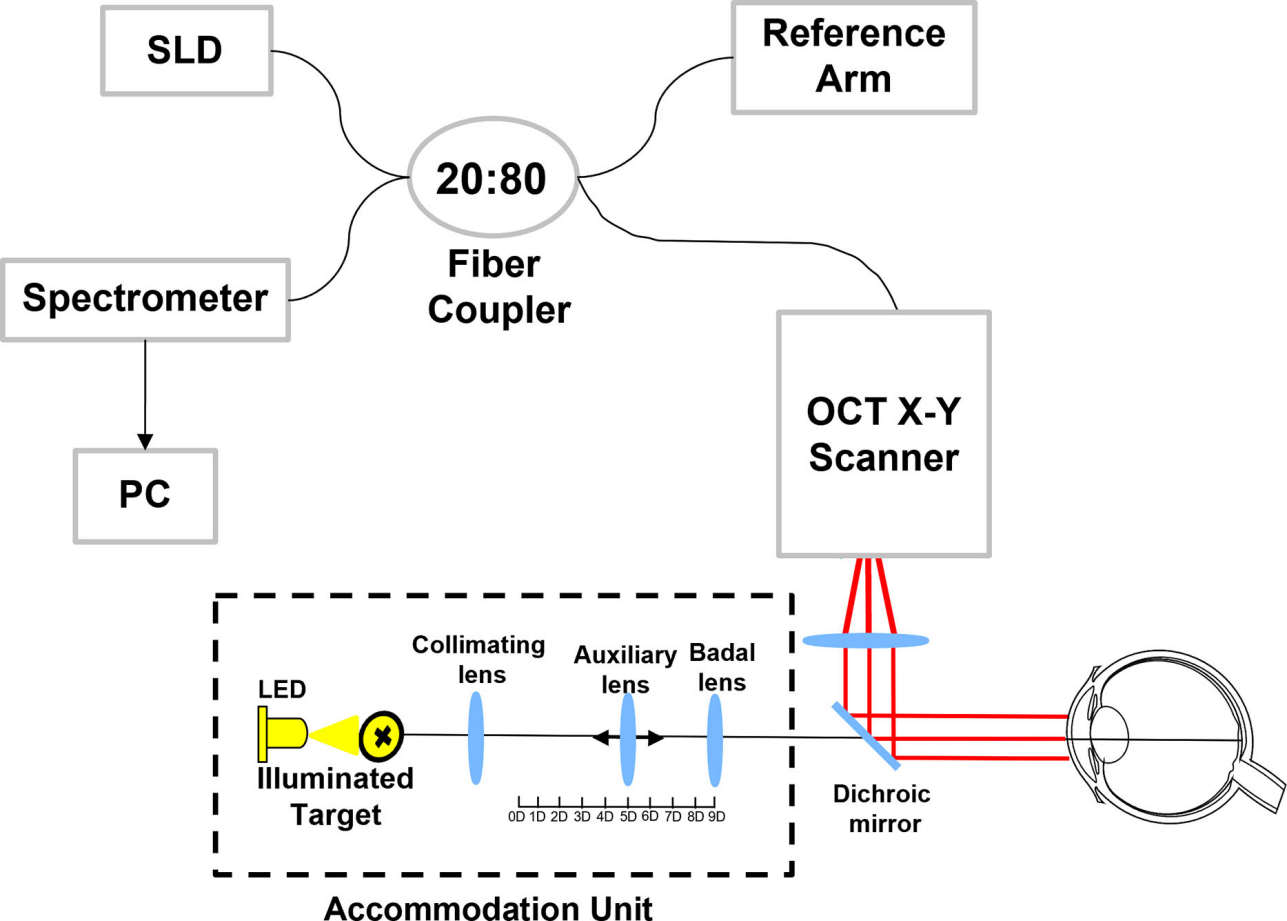
Schematic of the spectral domain OCT system with the accommodation unit.

The imaging system used in this study was a custom-built extended-depth spectral domain OCT system operating at 840 nm with an axial resolution of 8 µm over an axial range of 10.5 mm in air. The OCT system was combined with a custom-built fixation target that provided an accommodation stimulus with adjustable vergence (Fig. 1).^12^ The images acquired from the OCT consisted of 2048 pixels in depth. During the image acquisition, the participant’s photoplethysmograph (PPG) was simultaneously measured at a sampling rate of 50 Hz using a pulse oximeter (PulseSensor; World Famous Electronics LLC, Brooklyn, NY, USA) clamped to the participant’s left index finger. A microcontroller-based electronic circuit was used to synchronize image and PPG acquisition.

### Experimental Protocol

The experiment was conducted with the room's lights turned off in a dark room under natural pupil conditions with the sole illumination of the accommodation target. Under these lighting conditions, the contralateral eye was not patched, but participants who felt the need to cover their contralateral eye were able to do so with a handheld eye occluder. The participant was seated in front of the device, and we stabilized the participant's head position by using a head and chin rest. No bite bar was used. Then, the pulse oximeter was clamped to the participant’s left index finger. Next, we instructed the participant to fixate the fixation target with the left eye. The vergence of the fixation target was first adjusted to the participant’s far vision (relaxed accommodative state). Then, we determined the maximal accommodation demand by adjusting the vergence in 0.5-D steps until the participant could no longer focus on the target. The fixation target was then brought back to the distance position, and we began the image acquisition. All the acquisitions were made in the accommodating eye. We aligned the OCT system with the eye's optical axis by visualizing a real-time image and adjusting the position using the joystick.^12^ The image acquisition was then started. We recorded the central 2-mm zone of the lens with 170 A-lines/frame and 50 frames/s for 10 seconds (500 frames per participant per accommodation demand). We synchronously recorded the participant's PPG at 50 Hz using the pulse oximeter. Recordings were acquired with the vergence set at 0 D, 2 D, 4 D, and maximal accommodation for young adults and 0 D and maximal accommodation for prepresbyopes. The measurements were repeated four times for each vergence before moving to the next vergence.

### Data Processing and Analysis

**Figure 2.**
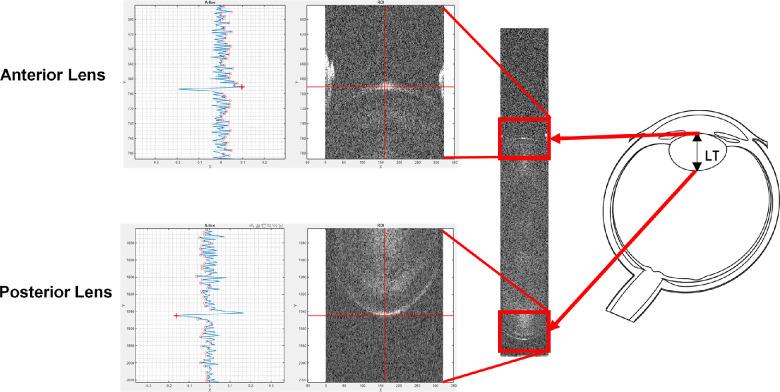
Image of the lens with the posterior lens and anterior lens surface selected as the region of interest in the *red rectangle*. On the *left*, the gradient profile alongside with the selected region. The position of the surface is saved for each image. Both surfaces are segmented separately.

A custom-designed semiautomated MATLAB application (MathWorks, Natick, MA, USA) was used to detect the anterior and posterior lens in each image and calculate the lens thickness. The measurement relied on peak detection in the gradient of the average intensity profile of the 40 A-lines covering the central zone of the lens (Fig. 2). The optical thickness of the lens was divided by the average group refractive index (*n* = 1.415) to produce the geometrical thickness.^13^ We then subtracted the mean from the time trace of the lens thickness variation to remove the DC component and then performed a Fourier transform to obtain the power spectrum of the lens thickness microfluctuations. The amplitude of the microfluctuations was defined as the area under the power spectrum in the frequency band ranging from 0 to 4 Hz. The integration was limited to 4 Hz because a preliminary analysis showed that there was no detectable signal at frequencies beyond 4 Hz. We also quantified the fluctuations in the two spectral bands defined in prior studies, LFC (0–0.6 Hz) and HFC (1.0–2.5 Hz). For each participant, we calculated the average amplitude of the microfluctuations across the four trials. By quantifying the microfluctuations in the spectral domain, we were able to capture information about the frequency distribution of the lens thickness microfluctuations, particularly the evolution of LFC and HFC spectral bands defined by prior studies.

We calculated the power spectrum of the PPG trace to determine the arterial pulse frequency. We then verified whether there is a frequency peak in the power spectrum of the lens microfluctuations corresponding to the arterial pulse frequency. Additionally, we calculated the cross-correlation of the time-dependent PPG signal and lens thickness fluctuations to determine if lens thickness fluctuations are associated with the arterial pulse. To account for any potential delay in the effect of the arterial pulse on lens thickness, we calculated the cross-correlation after introducing a shift between the PPG and lens thickness signals ranging from −0.5 to +0.5 seconds in 0.05-second steps.

We used an independent *t*-test to determine if there are significant differences in the lens thickness microfluctuations between the two independent age groups, while we used a paired *t*-test to determine if there are significant differences between accommodative states in the same age group. We also used a paired *t*-test on the averaged LFC and HFC amplitude values of each participant for each group (young and prepresbyope) to determine if there is a significant difference between the amplitude of LFC and HFC. We set the significance level at *P* = 0.05.

To assess the participant’s objective accommodative response, we calculated the average and standard deviation of the lens thickness of each recording. We also visually inspected each lens thickness recording to ensure that there were no drifts or discontinuities reflecting a loss of fixation or inability to maintain steady accommodation. In such cases, the data were discarded, and we recorded a new measurement, except in three young participants and one prepresbyopic participant in whom the measurements could not be repeated. The following measurements were discarded in these four participants (see Supplementary Data Table): two of four measurements recorded at 2 D and one of four at 4 D in young participant no. 2, two of four measurements recorded at 4 D in young participant no. 4; one of four measurements recorded at 2 D in young participant no. 5, and one of four measurements recorded at maximum accommodation for prepresbyopic participant no. 4.

## Results

**Figure 3.**
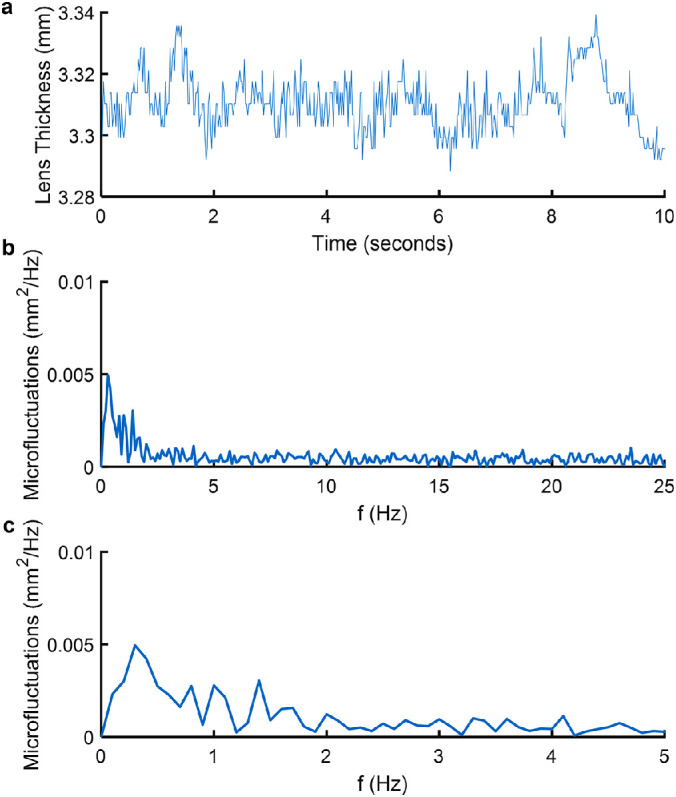
(**a**) Typical trace of the lens thickness variation of a 20-year-old participant at relaxed state (0 D accommodation). (**b**) Power spectrum of the lens thickness fluctuations from 0 to 25 Hz and (**c**) zoomed-in version from 0 to 5 Hz of the power spectrum shown in (**b**).

**Figure 4.**
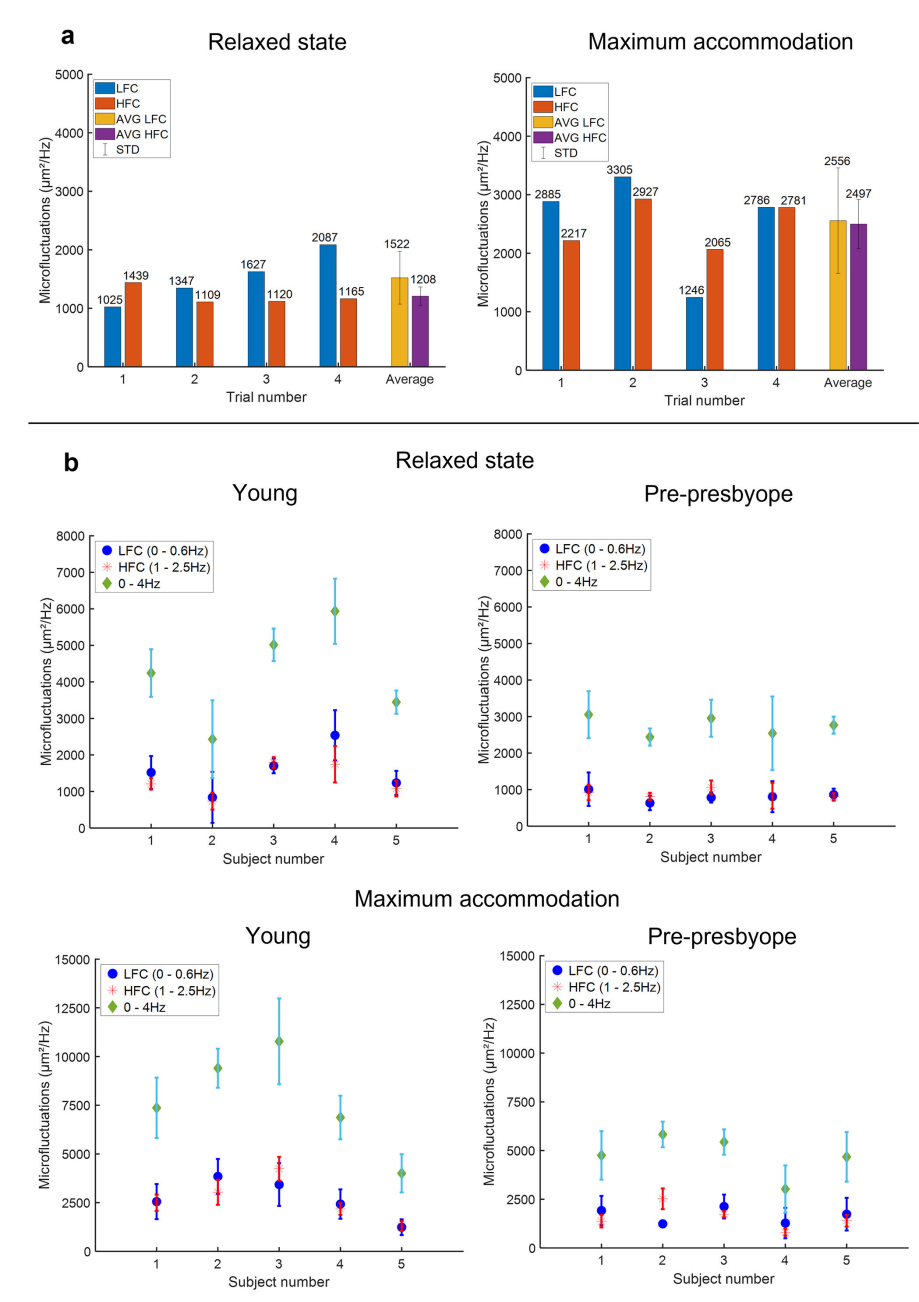
(**a**) LFC (0–0.6 Hz) and HFC (1.0–2.5 Hz) of the lens thickness microfluctuations for a 26-year-old participant. (**b**) Average and standard deviation of the LFC, HFC, and microfluctuations from 0 to 4 Hz of the four trials for the 10 participants for relaxed and maximal accommodated state. There is no statistically significant difference between the LFC and HFC components in the relaxed (*P* = 0.16 for young participants and *P* = 0.38 for prepresbyopes) and accommodated states (*P* = 0.86 for young participants and *P* = 0.87 for prepresbyopes). Note the different scaling in the different plots.

**Figure 5.**
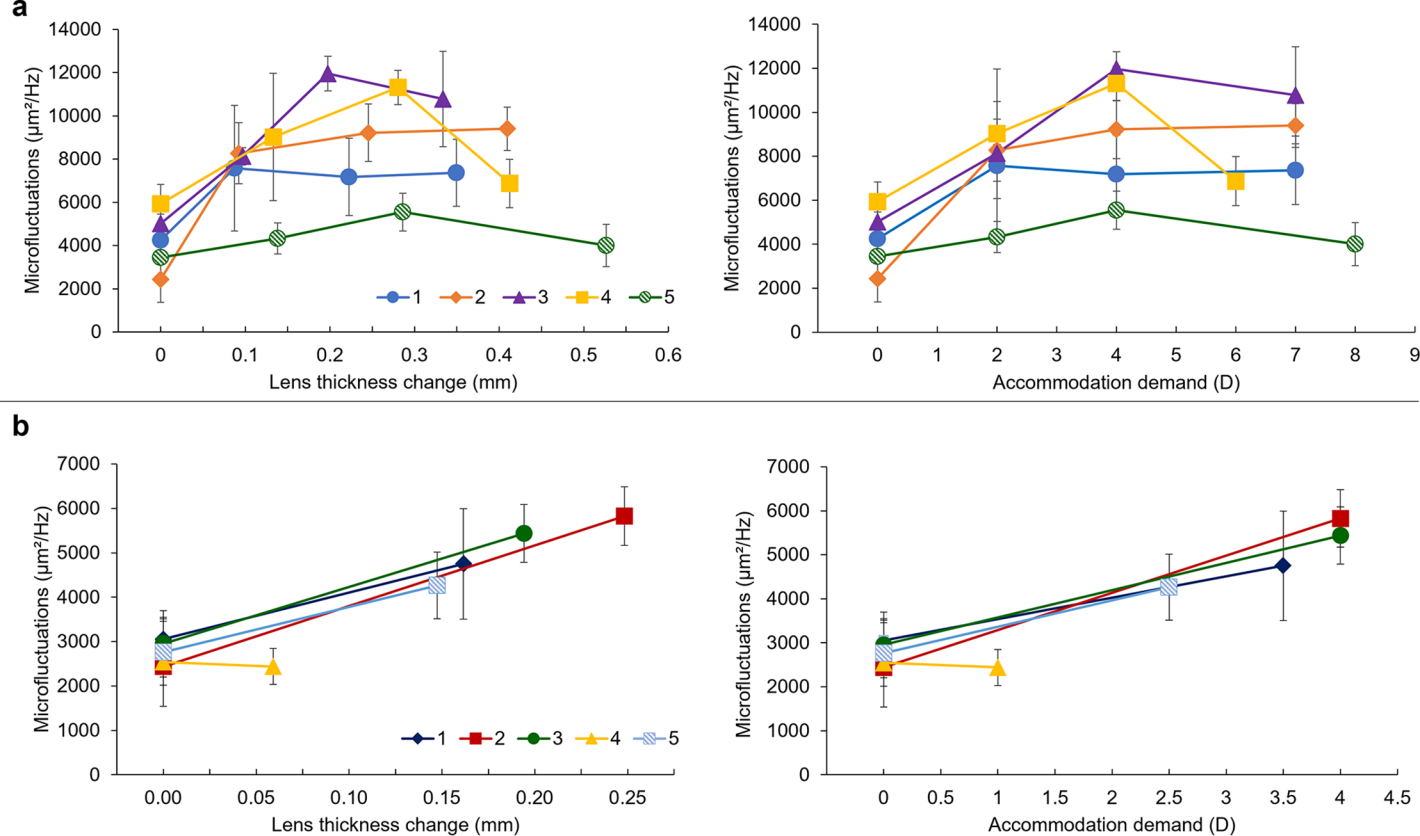
Evolution of the lens thickness microfluctuations of the (**a**) five young and (**b**) five prepresbyopic participants in terms of lens thickness change and accommodation demands. Note the different scaling in the different plots.

**Figure 6.**
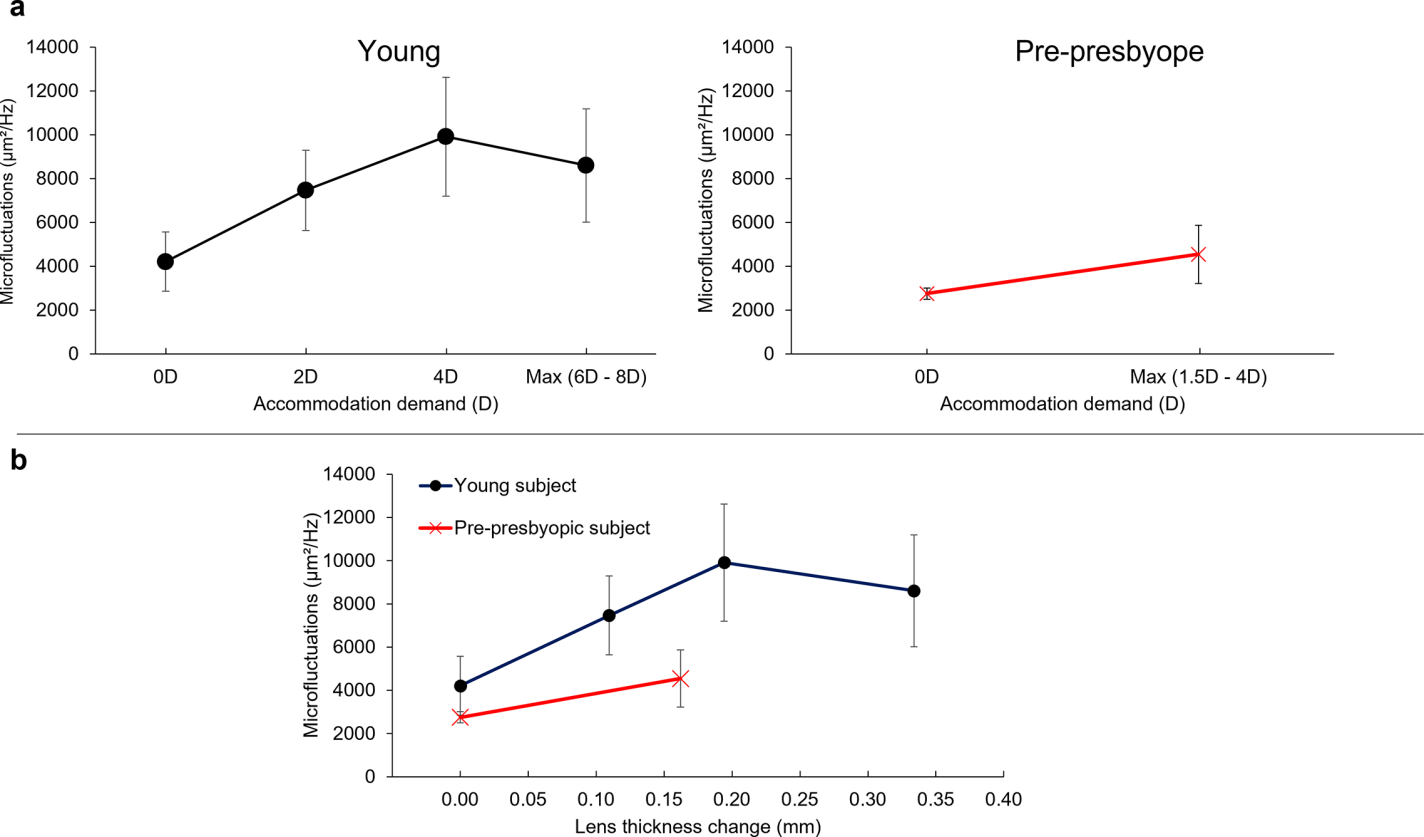
Evolution of the five young and five prepresbyopic participants’ averaged lens thickness microfluctuations in terms of (**a**) accommodation demand and (**b**) lens thickness change.

The maximal accommodation demand was 7.0 ± 0.7 D (range, 6–8 D) for young participants and 3.0 ± 1.3 D (range, 1–4 D) for prepresbyopes. One of the prepresbyopes demonstrated a minimal response (0.06-mm thickness change, participant 4). Figure 3 illustrates the lens thickness microfluctuations in a young participant at relaxed state and their power spectrum. We could not distinguish any predominant discrete frequency peaks. We found that there is no statistically significant difference between the averaged amplitude of the LFC and HFC in young (*P* ranging from 0.16 to 0.86 depending on accommodation demand) and prepresbyopic participants (*P* = 0.39 for relaxed state and *P* = 0.87 for maximum accommodation) at any of the accommodative states. In individual participants, we also found that there is no statistically significant difference between the amplitude of the LFC and HFC in any accommodative state, except for one prepresbyope at maximum accommodation (Fig. 4). In both groups, the lens thickness microfluctuations were greater when the lens was accommodated than when it was relaxed (Fig. 5). For the young participants (Fig. 5a), the increase was neither linear nor monotonic with accommodation. Microfluctuations reached a plateau as the eyes accommodated or a peak followed by a decrease as the participant reached maximal accommodation. There was no statistically significant difference between the amplitude of microfluctuations at the two last accommodative states in four of five participants (*P* ranging from 0.16 to 0.95). On average (Fig. 6), we found that the amplitude of lens thickness microfluctuations increased up to the 4 D accommodation demand and then plateaued.

Lens thickness microfluctuations were larger in the young participants than in the prepresbyopic participants at both relaxed (*P* = 0.04) and maximal (*P* = 0.04) accommodation (Fig. 6b). The difference was more pronounced in the maximum accommodative state than in the relaxed state. To verify that the difference between young and prepresbyopic participants was not due to differences in accommodative responses with age, we could compare the amplitude of microfluctuations at equal lens thickness change (i.e., for the same objective accommodative response). For an equivalent change in thickness, young participants still showed higher fluctuations than prepresbyopic participants (Fig. 6b).

**Figure 7.**
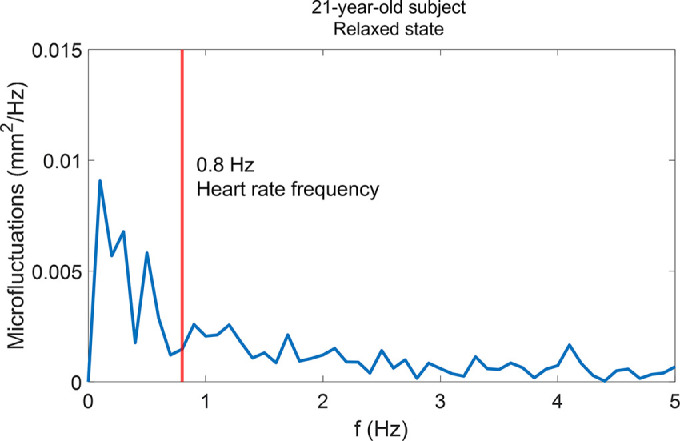
Power spectrum of the lens microfluctuations of a 21-year-old participant in the relaxed state (0 D accommodation). The *vertical red line* is the heart rate frequency (0.8 Hz).

**Figure 8.**
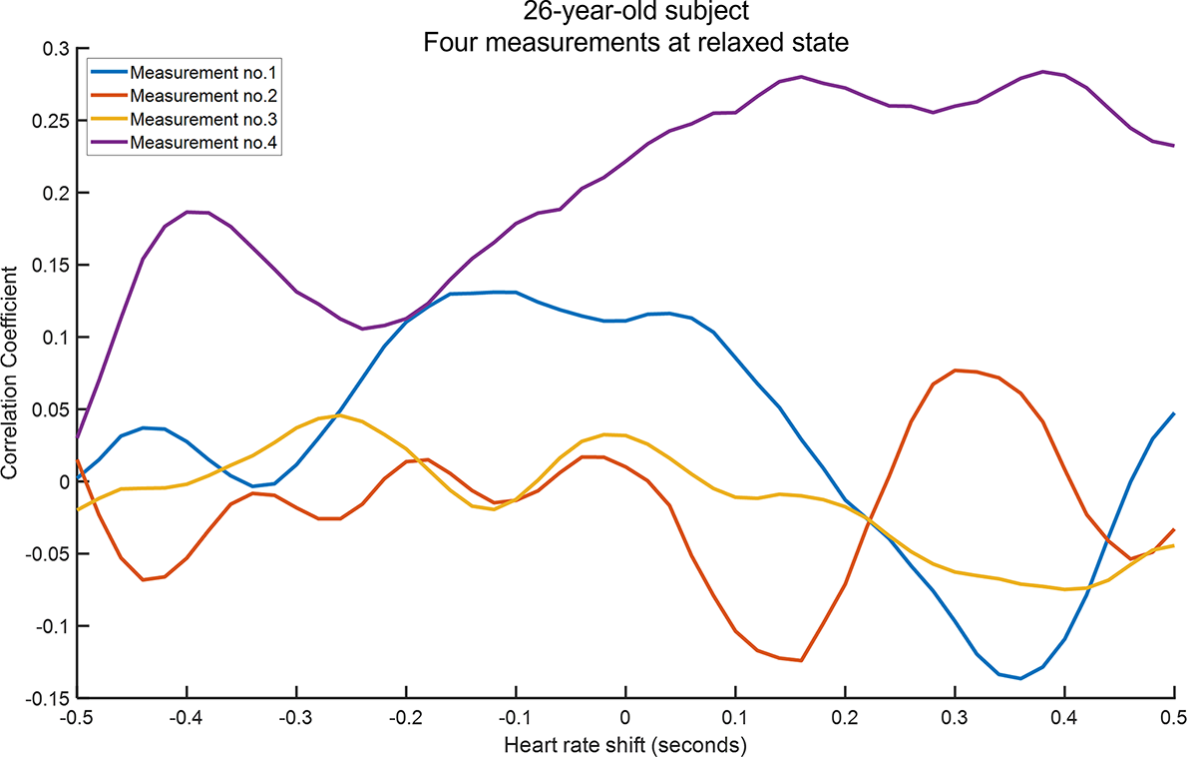
Cross-correlation between lens thickness microfluctuations and PPG trace for a 26-year-old participant at relaxed state (0 D accommodation). The different behavior observed for the fourth measurement is due to differences in the amplitude of the PPG signal and lens microfluctuations.

We found no correlation between the lens microfluctuations and the PPG trace (Fig. 7 and Fig. 8). No distinct peak was found in the cross-correlation plot at any of the shift values (Fig. 8).

## Discussion

This study aimed to determine if the mechanical microfluctuations of the lens measured using high-speed OCT biometry are dependent on age and accommodation demand. We observed that lens thickness microfluctuations are more prominent in young adults than in prepresbyopes. We also found that lens thickness microfluctuations are higher in the accommodated state than in the relaxed state. The dependence on accommodation demand, however, is neither linear nor monotonic. A further observation of this study is the absence of a distinct peak in the HFC frequency band (1.0–2.5 Hz), consistent with the earlier findings of Van der Heijde et al.^10^ In addition, we found no relationship between lens thickness microfluctuations and arterial pulse.

Prior studies on the optical microfluctuations of accommodation generally quantify the amplitude separately in the LFC and HFC frequency bands following the observation that there are discrete signal bands or peaks in these frequency ranges.^1^^,^^7^^,^^14^^–^^16^ A few studies quantified the optical microfluctuations over the entire frequency band of the recording.^7^^,^^14^^,^^17^^,^^18^ Since we did not observe any distinguishing features or discontinuities in the power spectrum in the LFC and HFC bands, we integrated the spectrum from 0 to 4 Hz. We used 4 Hz as the upper limit, instead of calculating the total integrated spectral amplitude from 0 to 25 Hz because we did not find any detectable signal component beyond 4 Hz. When we analyzed our results using the traditional LFC and HFC bands, we found that there is no statistically significant difference between amplitude of the lens microfluctuations in the two bands. Instead, our results suggest that the frequency spectrum of the lens thickness microfluctuations is characterized by a strong low-frequency component with an amplitude that decays continuously and reaches the noise level at frequencies around 2.5 to 3 Hz. The strong low-frequency component observed in all participants may have been caused by a slow drift of accommodation or fixation. To eliminate the potential contribution of drifts, we performed a separate analysis where we excluded the very low-frequency band from 0 to 0.3 Hz. This separate analysis produced the same general findings in terms of accommodation and age. Overall, our results suggest that the HFC component found in previous studies of optical microfluctuations of accommodation does not have a lenticular origin. It may instead be caused by fluctuations in axial eye length as observed by Van der Heijde et al.^10^

To ensure that the lens thickness microfluctuations that we are measuring are not caused by eye movements or microsaccades, we simulated the effect of lateral displacements and microsaccades on the lens thickness measurement using ray-tracing in a four-surface eye model. We assumed that the center of rotation of the eye is located 15 mm behind the cornea, typical for a human eye. We calculated the lens thickness microfluctuations for microsaccades with an amplitude of 0.5°, based on prior studies showing that 90% of saccades are below 31 arcmin (0.52°).^19^ The ray-tracing simulation shows that with the thickest and most curved lens shape (worst-case scenario), the lens thickness change is only 3.3 µm, below the 5-µm digital resolution of our imaging system, and therefore not detectable in our measurements. For a 1° saccade, the change in thickness is only 13 microns (less than 3 pixels), well below the amplitude of the fluctuations measured in our studies. The simulation also shows that a lateral movement of 0.2 mm is required to produce a lens thickness change of 5 microns (1 image pixel). This analysis shows that the lateral or rotational eye movements that would be required to produce the measured lens thickness fluctuations would be detectable in the OCT images. However, as it can be seen in the real-time video in Figures 9a and 9b, a deviation of the lens is not visible. Moreover, it has been shown that saccades have a broad frequency spectrum, well above the 4 Hz bandwidth that we detect in our recordings, and that a higher speed sampling system is necessary to measure eye saccades.^20^ An imaging system operating at 50 Hz cannot reliably detect saccades smaller than 5°.^21^ If saccades had had an effect on our measurements, we would have observed a much broader frequency spectrum. We are therefore confident that the lens thickness fluctuations that we are measuring are not due to eye movements.

**Figure 9a.**
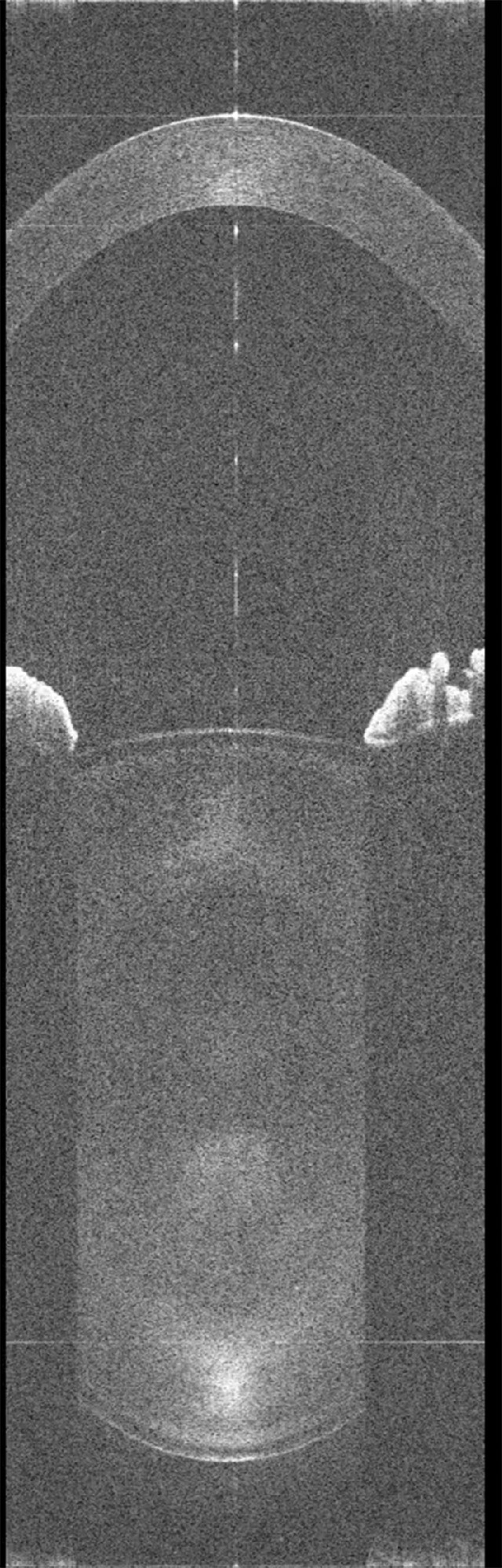
Real-time video of the anterior segment of pre-presbyopic participant 1 at relaxed state during a 10-second acquisition. Movies are available on the journal website.

**Figure 9b.**
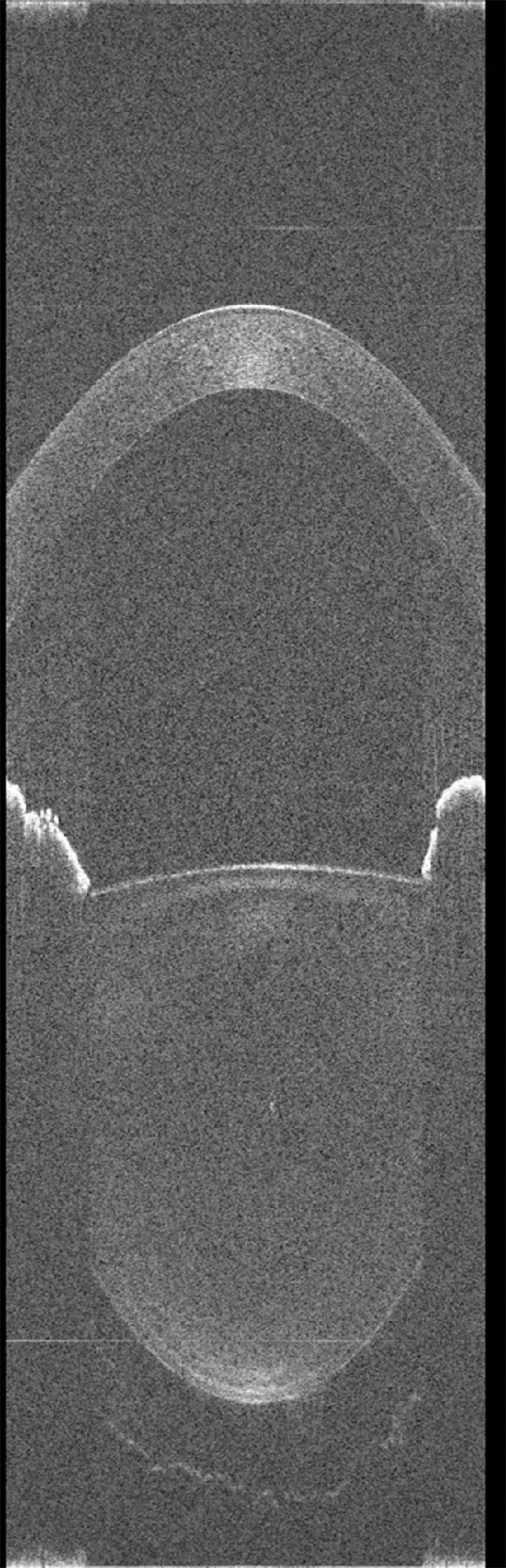
Real-time video of the anterior segment of young adult participant 4 at relaxed state during a 10-second acquisition. Movies are available on the journal website.

We found that lens thickness microfluctuations decreased with age, in agreement with previous studies showing that ocular dioptric microfluctuations decrease with age.^17^^,^^22^^–^^24^ Lens thickness microfluctuations at steady state of accommodation are most likely caused by fluctuations in zonular tension that cause a fluctuation in the force applied to the lens. The finding that lens thickness microfluctuations decrease with age provides support to the hypothesis that the decrease in optical microfluctuations of accommodation with age is due to changes in lens viscoelasticity.^2^^,^^17^^,^^22^ However, changes in the fluctuations of zonular tension with age due to age-related changes in lens shape, ciliary body geometry, or zonular elasticity could also contribute to the age dependence of lens thickness microfluctuations. Further studies are needed to quantify the respective contribution of these potential factors to the age-related decrease in lens thickness microfluctuations.

Our finding that lens thickness microfluctuations increase with accommodation confirms previous studies that characterized the changes in optical microfluctuations in terms of accommodation demand.^4^^,^^7^^,^^23^^,^^25^ It is also consistent with the findings of Gambra et al.^11^ However, Gambra et al.^11^ found that this increase is due primarily to an increase in the HFC, while we do not observe a statistically significant difference between the LFC and HFC. We further observed that, on average, the lens thickness microfluctuations reach a plateau as the participant nears the maximum accommodation after an initial linear phase of increase. Previous studies produced inconsistent results regarding the change with accommodation. Toshida et al.,^23^ for example, did not measure the LFC but found that the HFC increases up to an intermediate vergence and then decreases at higher vergences. Studies that quantified both the LFC and HFC found that only the LFC increased with accommodation.^10^^,^^26^ Finally, studies that quantified the entire power spectrum without differentiating between the LFC and HFC found a maximum of optical microfluctuations of accommodation at an intermediate vergence.^7^^,^^14^^,^^27^ We find that both the LFC and HFC of lens thickness microfluctuations increase with accommodation in agreement with our observation that the power spectrum of lens thickness microfluctuations is a continuous function over the spectral band containing the LFC and HFC.

The accommodation-dependent increase in lens thickness microfluctuations may be attributed to increased mechanical instability of the lens when the tension on the zonular fibers is released during accommodation.^16^^,^^18^^,^^23^ It could be due either to an increase in fluctuations of zonular tension or to an increase in the force produced by fluctuations of zonular tension. In the latter case, the findings would suggest that fluctuations in zonular tension produce larger fluctuations in lens shape as the tension on the zonule is released (i.e., when the eye is in accommodated state).

Similarly, we believe that the plateau or decrease in lens thickness microfluctuations observed as the lens reaches maximal accommodation reflects the increased resistance of the lens contents to the molding force of the lens capsule as the lens becomes maximally rounded, possibly limiting or reducing the amplitude of mechanical fluctuations.^28^ Additional studies using smaller vergence steps (0.5 or 1 D instead of 2 D in the current study) are needed to confirm these hypotheses.

We found no correlation between lens thickness microfluctuations and arterial pulse. Previous studies on the ocular dioptric microfluctuations of accommodation found that the HFC is correlated with arterial pulse.^8^^,^^9^ The authors of these studies hypothesized that the vascular pulsation in the ciliary muscle causes fluctuations in ciliary ring diameter and intraocular pressure, which causes changes in lens shape. The lack of correlation between arterial pulse and lens thickness microfluctuations in our study contradicts this hypothesis. Our results also confirm the findings of Van der Heijde et al.^10^ that the correlation between arterial pulse and microfluctuations of accommodation is not due to lens shape but may be due to vitreous depth.

Our study demonstrates the feasibility of quantifying lens thickness microfluctuations at high frame rates (50 Hz) using extended-depth OCT. The key advantages of OCT compared to ultrasonography are that it is a noncontact technique with higher axial resolution. Our axial resolution of 8 µm in air corresponds to a resolution of 5.7 µm in the lens, assuming a group refractive index of 1.415. This resolution is sufficient to accurately quantify lens thickness microfluctuations. Previous studies have found that lens thickness increases at a rate of 40 to 70 µm per diopter of accommodation demand^10^^,^^29^ and that the steady-state peak-to-peak amplitude of microfluctuations is within ±0.5 D,^17^^,^^18^ which corresponds to an approximate thickness change of 20 to 35 µm, well above the resolution of our OCT system.

In summary, we quantified the accommodation dependence of lens thickness microfluctuations in young adults and prepresbyopes as a function of accommodation. Our results show that the increase in ocular dioptric microfluctuations with accommodation found in prior studies is lenticular in origin and consistent with increased mechanical instability of the lens as the zonular tension is released. On the other hand, our results show that the origin of the high-frequency component of ocular dioptric microfluctuations is not lenticular.

## Supplementary Material

Supplement 1

Supplement 2

Supplement 3
